# Compensation of gene dosage on the mammalian X

**DOI:** 10.1242/dev.202891

**Published:** 2024-08-14

**Authors:** Daniela Cecalev, Beatriz Viçoso, Rafael Galupa

**Affiliations:** ^1^ Molecular, Cellular and Developmental Biology (MCD) Unit, Centre de Biologie Intégrative (CBI), University of Toulouse, CNRS, UPS, 31062, Toulouse, France; ^2^ Institute of Science and Technology Austria (ISTA), Am Campus 1, Klosterneuburg 3400, Austria

**Keywords:** X chromosome, X-chromosome inactivation, X-chromosome upregulation, Dosage compensation, Mammals

## Abstract

Changes in gene dosage can have tremendous evolutionary potential (e.g. whole-genome duplications), but without compensatory mechanisms, they can also lead to gene dysregulation and pathologies. Sex chromosomes are a paradigmatic example of naturally occurring gene dosage differences and their compensation. In species with chromosome-based sex determination, individuals within the same population necessarily show ‘natural’ differences in gene dosage for the sex chromosomes. In this Review, we focus on the mammalian X chromosome and discuss recent new insights into the dosage-compensation mechanisms that evolved along with the emergence of sex chromosomes, namely X-inactivation and X-upregulation. We also discuss the evolution of the genetic loci and molecular players involved, as well as the regulatory diversity and potentially different requirements for dosage compensation across mammalian species.

## Teaser

**Summary:** This Review focuses on sex-chromosome dosage compensation in mammals, discussing classic and recent hypotheses, observations and mechanisms regarding X-chromosome upregulation and X-chromosome inactivation.

## Introduction

Gene dosage denotes the number of gene copies present in a cell of an organism, which can be reflected in the amount of gene products, such as proteins and functional RNAs (Basilicata and Keller Valsecchi, 2021). Changes in gene dosage can, therefore, produce significant phenotypic consequences. For an individual, they often lead to harmful consequences (e.g. gene amplification of HER2 receptor associated with breast cancers; Seshadri et al., 1989) but, at an evolutionary scale, they contribute to adaptation and speciation (e.g. gene duplications; Kondrashov, 2012; Qian and Zhang, 2014). Gene-dosage changes can arise due to copy number variations (e.g. gene amplification, insertions or deletions) or changes in ploidy (i.e. the number of chromosomes sets in a cell via gain or loss of chromosomes or whole-genome duplications). The effects of having an extra chromosome are a lot more detrimental than having a whole extra set of chromosomes, as initially revealed by seminal experiments with the flowering plant *Datura stramonium* and with *Drosophila melanogaster* (Blakeslee, 1934; Blakeslee and Belling, 1924; Blakeslee et al., 1920; Bridges, 1921; reviewed by Birchler and Veitia, 2021). This led to the ‘gene-balance hypothesis’ (Birchler and Veitia, 2007, 2010, 2012), whereby maintaining a balanced gene dosage across the genome is crucial, especially for genes coding for products involved in functions where stoichiometry is important (e.g. members of multi-subunit complexes). Sex chromosomes (also known as ‘allosomes’) challenge such balanced gene dosage (Graves, 2006). In many animal and plant species where sex is determined by chromosomes (Box 1), individuals within the same population naturally exhibit differences in copy number for the genes within the sex chromosomes. In mammals, which have an XY sex-determination system and are the focus of our article, females have two copies of each gene residing on the X chromosome(s), whereas males have only one such copy, plus one copy of Y-linked genes, many of which are ‘unmatched’ in females (Box 2). According to GENCODE, the X chromosome in mouse has at least 932 protein-coding genes and 558 noncoding-RNA genes (Frankish et al., 2023); therefore, such asymmetry in gene dosage could lead to significant phenotypic consequences if left uncompensated. The X harbours genes involved in fundamental cell processes independent of sex-related functions; dozens of housekeeping genes are found on the X of both humans and mice: 99 and 91, respectively, according to a recent database (Hounkpe et al., 2021). This is consistent with mammalian sex chromosomes evolving from a precursor pair of autosomes, as we review below. The asymmetry in X-linked gene dosage in relation to autosomal gene dosage between the two sexes is thus thought to have favoured the emergence of sex-specific ‘dosage-compensation’ strategies.

**Box 1. Non-mammalian sex-chromosome systems and dosage compensation**

Sex chromosomes evolved independently in many animals and plants, and consequently so did dosage compensation (DC). In *Drosophila*, the RNA-protein complex used for DC is only assembled in males. It targets and opens the chromatin of the male X chromosome through the addition of the histone modification H4K14ac, leading to the doubling of transcription from this chromosome (Copur et al., 2018). A similar, convergent mechanism has been reported for a lizard species (Marin et al., 2017) and monarch butterflies (Gu et al., 2019). In the mosquito *Anopheles gambiae*, the male X chromosome is upregulated through a DNA-binding factor that is male-specific, as recently described (Kalita et al., 2023); interestingly, lack of DC is compatible with life in this species. The nematode *Caenorhabditis elegans* uses a more complex mechanism that, similar to mammals, likely involves the upregulation of some X-linked genes (Lau et al., 2016), combined with the global downregulation of expression of both X chromosomes in XX hermaphrodites (Meyer, 2022). Despite their different modes of action, both mechanisms work through the modulation of the chromatin landscape of the X chromosome (Jordan et al., 2019), a pattern that has now been suggested also in Lepidoptera (moths and butterflies), which have nematode-like compensation (Huylmans et al., 2017; Rosin et al., 2022; Tomihara et al., 2022; Yoshido and Marec, 2023) and in the plant *Silene latifolia*, which shows a form of X-inactivation reminiscent of mammalian DC (Lorenzo et al., 2020; Muyle et al., 2022). Surprisingly, given the essentiality of DC in model organisms, several species balance only a subset of genes, lacking a chromosome-wide mechanism of DC [‘gene-by-gene’ DC (Mank et al., 2011)]. This is often the case in species with female-heterogamety (males have two Z chromosomes, females are ZW), such as birds and snakes, where most Z-linked genes are expressed at lower levels in ZW females than ZZ males (Gu and Walters, 2017; Julien et al., 2012; Marin et al., 2017). Why balancing a subset of genes is sufficient in some species, whereas complex chromosome-wide mechanisms evolved in others, remains an open question.

**Box 2. The common genes between the X and the Y**

Although many genes on the Y chromosome were lost and others acquired *de novo* (and thus male-specific), a subset is still shared with the X chromosome. Many of these are located in the ‘pseudoautosomal region’ (PAR) of the sex chromosomes, which recombines during meiosis like autosomes and is identical between the X and the Y. These genes, biallelically expressed in XY individuals, escape XCI and are thus biallelically expressed in XX individuals as well (Navarro-Cobos et al., 2020) – dosage compensation is thus presumably achieved through escape. Interestingly, compared with the active X counterpart, the expression levels of the homologue gene on the Y or the escaping allele on the inactive X appear to be lower (Disteche et al., 2003). Other ancestral gene pairs retained on the X and the Y (e.g. *UTX*/*UTY*, *KDM5C*/*KDM5D*) reside outside of the PAR and therefore do not undergo meiotic recombination, resulting in fixed genetic differences between the X and Y homologues. Some of the X-linked counterparts, like *UTX* and *KDM5C*, are constitutive XCI escapees, which has been believed to be a means of dosage compensation, suggesting that the X-Y homologous pairs have retained common functions. However, recent studies have shown that, despite their homology and sometimes high sequence identity (>95%), the pairs can show striking functional differences. For example, *DDX3X* and *DDX3Y* exhibit unique biochemical properties primarily influenced by differences in their intrinsically disordered region 1, which significantly impact RNA metabolism and stress response, contributing to sex-biased susceptibilities observed in human diseases (Shen et al., 2022). Another example is the *NLGN4X*/*NLGN4Y* pair: both are expressed in the human brain but display significant functional differences due to a single amino acid variation affecting their cellular localisation and function in neurons (Nguyen et al., 2020). These findings prompt intriguing questions about the extent of functional gene content divergence between the X and Y chromosomes and its significance for dosage compensation. How do these differences contribute to the evolutionary adaptation of sexes (Martínez-Pacheco et al., 2020)? What impact do they have on susceptibility to sex-biased diseases and conditions (DeCasien et al., 2023)? Typically, escape genes that have Y homologues are believed to be under strong purifying selection, i.e. harmful mutations in these genes are less likely to be passed on, ensuring that the genes remain functional over generations, which highlights their important roles (Park et al., 2010). This is further evidenced by the fact that many genes associated with Turner syndrome (a condition involving the loss of one X chromosome in XX individuals) have counterparts on the Y chromosome (see references in Park et al., 2010).

## The emergence of sex chromosomes in mammals and their asymmetries in gene dosage

Susumu Ohno, a pioneer in the study of sex chromosome evolution, proposed that sex chromosomes originated from a precursor pair of autosomes (called ‘proto-sex chromosomes’), which underwent key mutations generating sex-determining loci (Ohno, 1967). Comparative genomics has revealed that the sex chromosomes of living mammals, which include prototherians (monotremes, such as the platypus), metatherians (marsupials, such as the wombat) and eutherians (placental mammals, such as the mouse), have different evolutionary origins (Bellott et al., 2014; Bininda-Emonds et al., 2007; Cortez et al., 2014; Luo et al., 2011; Marshall Graves, 2008; Messer et al., 1998; Potrzebowski et al., 2010). The sex chromosomes of marsupials and placental mammals are homologous, positioning their emergence approximately 166 million years ago (preceding the divergence of the metatherian and eutherian lineages), whereas the sex chromosomes of monotremes emerged through a parallel path (Box 3).

**Box 3. Sex chromosomes and dosage compensation in monotremes**

The platypus, an egg-laying mammal (Monotremata), has a peculiar set of sex chromosomes, consisting of five different X and five different Y chromosomes – platypus females are X_1_X_1_X_2_X_2_X_3_X_3_X_4_X_4_X_5_X_5_ and males are X_1_Y_1_X_2_Y_2_X_3_Y_3_X_4_Y_4_X_5_Y_5_ (Grützner et al., 2004; Rens et al., 2004; Warren et al., 2008; Zhou et al., 2021). The X_1_Y_1_ pair shows the highest similarity between each other, whereas the X_5_Y_5_ pair is the most divergent, suggesting the first pair is the evolutionarily youngest, while the latter is the oldest. The X_5_ chromosome harbours the gene *DMRT1*, involved in sex determination in birds (Chue and Smith, 2011; Ioannidis et al., 2021), implying a shared history with the bird Z chromosome and hinting at a ZW sex-chromosome system in ancestral mammals before the transition to the XY system. Dosage-compensation mechanisms in the platypus also share features with those in birds; an early study looking at individual genes revealed partial and variable dosage compensation (Deakin et al., 2008), meaning that some genes showed compensation and others did not, and this also depended on the tissue analysed. Omics analyses (Julien et al., 2012; Marin et al., 2017) showed signs of partial (∼1.5-fold) but global XCU in males (compared with ancestral levels), whereas in females expression levels seem unchanged. The partial dosage compensation in males has probably rendered the evolution of global XCI in females unnecessary (XCI is absent in monotreme females). Recently, unbalanced mRNA levels of X-linked genes have been confirmed (Lister et al., 2023 preprint), while quantification of protein abundance revealed balanced levels. This was, however, assessed for only a small fraction of the proteome (∼5%), so it remains an open question whether there are post-transcriptional mechanisms of dosage compensation in the platypus.

Ohno's hypothesis expanded on the idea of Hermann J. Muller that the differentiation of sex chromosomes would follow the lack of recombination caused by the appearance of a sex-determining gene (Muller, 1914). In therian mammals, the first step in the evolution of the proto-sex chromosomes is considered to be the acquisition of the male-determining gene, sex-determining region Y (*SRY*), on one of the proto-sex chromosomes (Foster and Graves, 1994; Foster et al., 1992; Gubbay et al., 1990). *SRY* is thought to have evolved from a mutation in one allele of the proto SRY-related HMG box-containing gene 3 (*SOX3*) gene (Collignon et al., 1996; Stevanović et al., 1993; Sutton et al., 2011). In present-day therian chromosomes, *SRY* is located on the Y chromosome and *SOX3* on the X chromosome. The emergence of *SRY* is thought to have been followed by a series of other events, the order of which remains under debate: the emergence/accumulation of other male-specific genes on the proto-Y, the suppression of meiotic recombination between the evolving proto-X and proto-Y chromosomes, and the progressive degradation of the proto-Y in terms of gene content due to lack of recombination (Bachtrog et al., 2011; Bergero and Charlesworth, 2009; Charlesworth et al., 2005; Chibalina and Filatov, 2011; Felsenstein, 1974; Rice, 1996; Wright et al., 2016). This progressive differentiation of the sex chromosomes meant that genes on the proto-sex chromosomes, once present in two copies and ‘in balance’ with genes across other autosomes, progressively became ‘haploid’ and (potentially) ‘unbalanced’. In other words, the heterogametic sex (XY) became a ‘natural aneuploid’ for X-linked genes (Disteche, 2016), with X-linked gene dosage reduced from two to one in XY individuals. This process is thought to have been accompanied by the emergence of dosage-compensation mechanisms to restore the balance between autosomal and allosomal gene expression, which we discuss below.

## Dosage compensating the X chromosome: a two-step hypothesis

Ohno's influential hypothesis on the evolution of the sex chromosomes (Ohno, 1967) put forth two steps to account for the dosage compensation of X-linked gene expression: a first step entailing an increase of the activity of the X chromosome, aiming to balance the levels of gene products from the single X in males with those from the two sets of autosomes; and a second step required to counteract the effects of the first one in females, by deactivating one of their two X chromosomes, thereby bringing the levels of X-linked gene products down to the disomic levels from autosomal chromosomes. This second step was drawn from the insightful hypothesis proposed by the geneticist Mary Lyon of X-chromosome inactivation (XCI), a phenomenon that has since been confirmed and is well-established (Blewitt, 2024; Lyon, 1961). The first step, on the other hand, presupposes the existence of X-chromosome upregulation (XCU), which has remained controversial in mammals.

### X-chromosome upregulation: hypotheses, observations and mechanisms

Longstanding controversies include whether or not XCU is present in mammals, and if so, whether it is global or affects only a subset of genes, and to which extent (whether it achieves complete dosage compensation or only partial). We have compiled a list of the studies that have investigated XCU in mammals and included their conclusions, approaches and data used for analysis (Table 1). One of the main reasons why different studies reached different conclusions is their approach when determining XCU. Many authors have compared the expression levels of X-linked genes with that of autosomal genes across several tissues in different mammals. Using this approach, the vast majority of studies have reported similar global levels of expression of X-linked and autosomal genes (based on expression ratios and/or distributions), thus concluding that upregulation of the single active X in mammals occurs. The two exceptions are a study using low-coverage proteomics data and a study in which non-expressed genes were not discarded from the analysis (Deng et al., 2011; He et al., 2011; Kharchenko et al., 2011). Concluding that XCU takes place from the fact that expression levels of the single active present-day X chromosome are similar to expression levels of autosomes assumes that, before sex-chromosome differentiation, expression levels of genes on the (ancestral) proto-sex chromosomes were similar to those on the ancestral autosomes. Such an assumption is not directly derived from Ohno's hypothesis, which did not postulate *similar* levels of expression but *balanced* levels of expression, which had to be preserved upon sex-chromosome differentiation. Thus, other authors have argued that comparing X-linked expression levels to autosomal expression levels is not a real test of Ohno's hypothesis (He et al., 2011; Julien et al., 2012; Lin et al., 2012). Instead, they should be compared to expression levels in the ancestral proto-sex chromosomes, for which these authors proposed to use, as a proxy, expression levels of the genes in monotremes and birds that are (autosomal) orthologs of the therian X-linked genes. Based on these comparative analyses, no upregulation was observed, leading the authors to refute Ohno's hypothesis. This approach makes assumptions too, and whether contemporary mammals can be directly compared to contemporary birds was initially questioned (Disteche, 2016). Meanwhile, consistent results have been achieved using other outgroups and various sets of autosomal genes (Julien et al., 2012; Marin et al., 2017; Wang et al., 2020), and although no XCU was found in placental mammals, full global XCU was demonstrated in marsupials (Julien et al., 2012). Current-to-ancestral comparisons may not always be feasible (if relevant data, including for outgroups, is not available; e.g. during specific developmental stages), so careful X-to-autosome comparisons can still be relevant and informative.

**Table 1.**
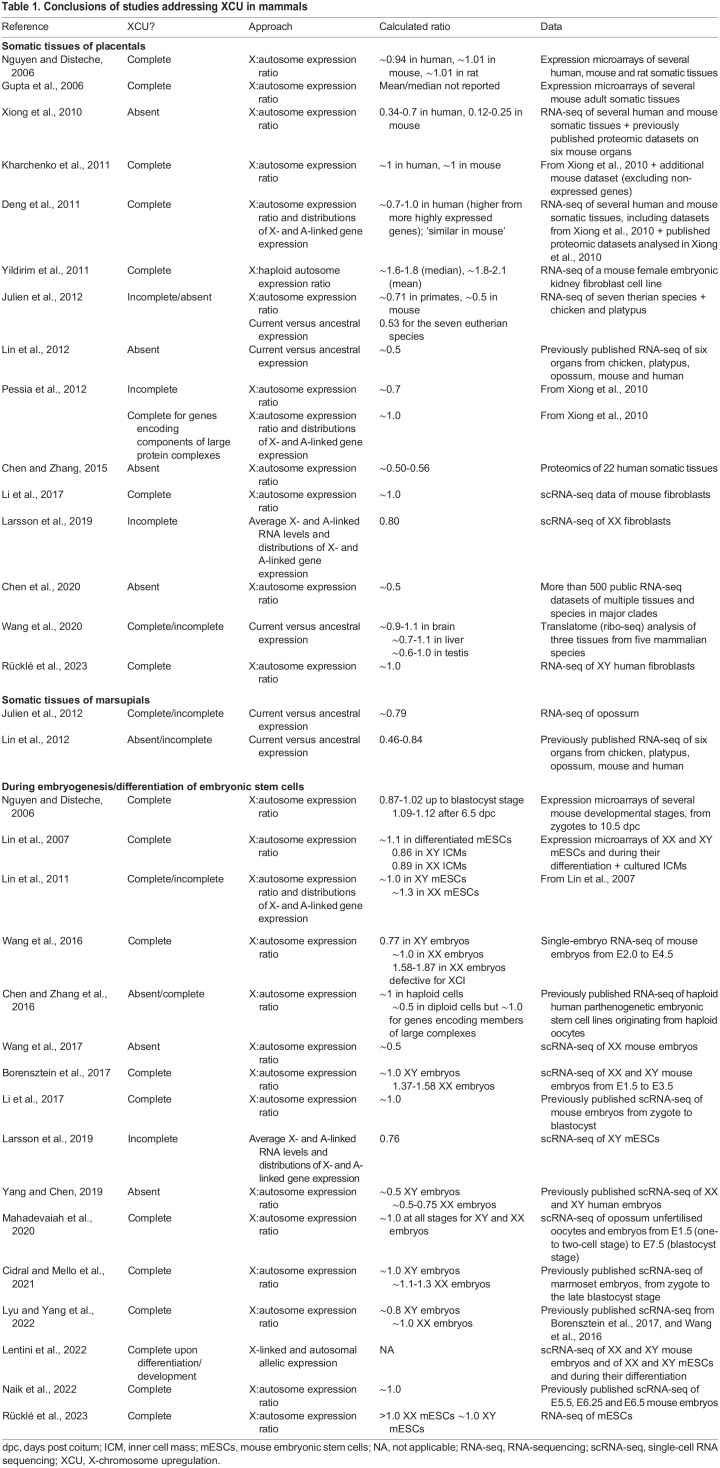
Conclusions of studies addressing XCU in mammals

These considerations mirror the challenge of defining sex-chromosome dosage compensation. Some authors adopt a broader definition, such as ‘the regulatory mechanisms that balance gene expression between the autosomes and sex chromosomes in the heterogametic sex’ (Mank et al., 2011), whereas others explicitly include the notion of the evolutionary history of the chromosomes; for example, ‘the maintenance of ancestral expression levels of sex-linked genes relative to autosomal expression in the heterogametic sex’ (Gu and Walters, 2017).

**Fig. 1.**
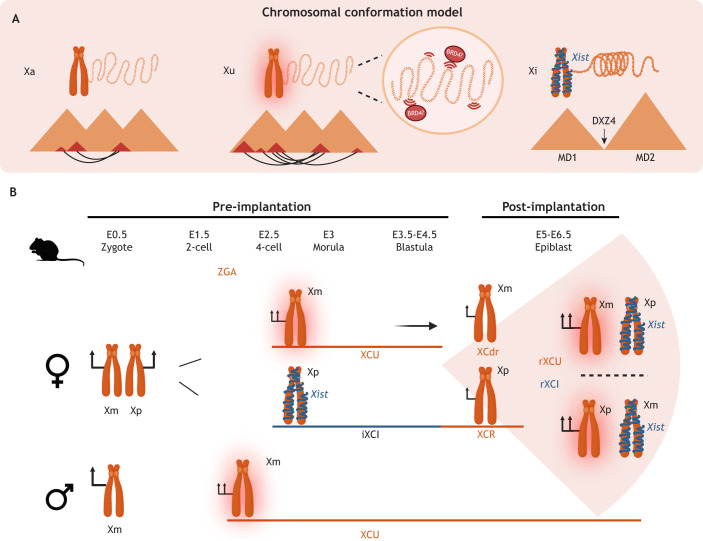
**Balancing the scales: the choreography of upregulation and inactivation of the X chromosome during mouse embryonic development.** (A) A hypothetical model of chromosome conformation dependent on the status of the X chromosome: active (Xa), upregulated (Xu), inactive (Xi). For the Xa state, an open chromatin configuration is illustrated, along with topologically associating domains (TADs) represented by orange and red triangles, and interactions between TADs shown as black connections. In the Xu state, it has been suggested that the number of interactions between TADs increases (Lentini et al., 2022), and there is ongoing debate regarding the potential involvement of BRD4 in transcriptional upregulation. In the Xi state, heterochromatin configuration is illustrated, along with the presence of the two megadomains (MD1; MD2) separated by the tandem repeat *DXZ4*. (B) Overview of the timing and dynamics during mouse pre- and early post-implantation development in both sexes. In XX embryos, from pre-implantation stages (E0.5-E4.5), the maternal X chromosome (Xm) undergoes upregulation (XCU) while the paternal X chromosome (Xp) is subject to inactivation (iXCI, *Xist* RNA in blue). At the blastocyst stage, Xm undergoes downregulation (XCdr), whereas Xp undergoes reactivation (XCR). Following this, in peri- to post-implantation stages, random XCI takes place, followed by (random) XCU on the other chromosome. In XY embryos, the sole X present (Xm) undergoes XCU immediately after ZGA, maintaining this upregulated state throughout development.

So, is there XCU or not in placental mammals? XCU as predicted in Ohno's hypothesis, which refers to higher expression of the present-day X compared with that of a single proto-sex chromosome, cannot be directly tested. As we reviewed, different approaches to address this question have different assumptions and have reached different conclusions. The question is not only whether the X is upregulated or not, but to what extent. Based on current-to-ancestral comparisons in placentals, a twofold upregulation is not achieved at the mRNA level but upregulation takes place – at least for some genes, as proposed by many (Naik et al., 2022; reviewed by Gu and Walters, 2017; Mank et al., 2011; Pessia et al., 2014). Recent single-cell, single-allele RNA-sequencing has confirmed higher expression from genes on the active X chromosome (Lentini et al., 2022), as discussed further below. At the molecular level, the (active) X chromosome is enriched in features that are all consistent with a ‘hyperactive’ transcriptional state compared with autosomes (Fig. 1). Its gene promoters show higher transcriptional burst frequencies (Larsson et al., 2019; Talon et al., 2021) and are enriched in the initiation form of RNA polymerase II, active histone marks, including histone acetylation (H4K16ac), and the corresponding acetyltransferase (MOF) that mediates XCU in *Drosophila* (Deng et al., 2011, 2013; Yildirim et al., 2011). Concomitantly, the active X shows higher chromatin accessibility than autosomes, as profiled by single-cell ATAC-seq (Talon et al., 2021). This investigation has identified increased chromatin accessibility on the active X chromosomes in mouse XX fibroblasts and XY mouse embryonic stem cells (mESCs), but not on the active X chromosomes of XX induced pluripotent stem cells (iPSCs) or mESCs. Interestingly, these results match the observations that the X chromosomes in mouse XX fibroblasts and XY mESCs are upregulated, whereas X chromosomes in XX mESCs are not (Larsson et al., 2019; Lentini et al., 2022). Recently, the BRD4 protein (containing bromodomains, which recognise acetylated lysine residues such as those in histones) has been implicated in the transcriptional activation of X-linked genes showing upregulation (Lyu et al., 2022), but this has been contested (Lentini and Reinius, 2023; Lyu et al., 2023).

A seemingly absent full dosage compensation (at the transcriptional level) has led to alternative hypotheses for the origin of XCI and partial XCU, unrelated to dosage compensation (Chandra, 1985, 2022; Engelstädter and Haig, 2008; Gribnau and Grootegoed, 2012; Haig, 2006; Iwasa and Pomiankowski, 2001; Mank et al., 2011; Pessia et al., 2014). Recently, however, the Kaessmann lab has proposed a reconciliatory perspective. Translatome analysis of tissues from four therian species (but not from platypus, a monotreme) revealed ‘translation upregulation’, with higher ratios of current-to-ancestral expression for the translatome than for the transcriptome (Wang et al., 2020). This was also associated with higher translation efficiencies and protein abundance (Wang et al., 2020). Combined with transcriptional upregulation, translational upregulation appears to have largely restored ancestral expression levels and, thus, X-to-autosome balance (Wang et al., 2020). Accordingly, previous studies have reported that X-linked transcripts have significantly higher ribosome density (Faucillion and Larsson, 2015) and longer half-lives (Deng et al., 2013; Faucillion and Larsson, 2015; Rücklé et al., 2023) than autosomal transcripts. Recently, depletion of RNA-associated N6-methyladenosine (m6A) modification led to a reduction in the ratio of X:autosome expression in both mouse and human cells, mainly through an increase in the stability of autosomal transcripts (Rücklé et al., 2023). X-linked transcripts were mostly unaffected, which is explained by their low(er) levels of m6A (Rücklé et al., 2023). This appears to be an intrinsic feature of X-linked transcripts, which show a depletion of the GGACH sequence, the m6A consensus motif (Rücklé et al., 2023). This suggests that the higher stability of X-linked mRNAs is hard-wired in the X-chromosome DNA sequence; how this has evolved remains an intriguing open question. In summary, full dosage compensation in placental mammals appears to be happening through a combination of transcriptional and post-transcriptional upregulation of gene expression (Wang et al., 2020), and thus Ohno's hypothesis stands after all.

Unlike XCU in *Drosophila* and XCI in mammals, mammalian XCU occurs in both sexes and does not appear to rely on a chromosome-wide mechanism – two aspects that we believe have contributed to hinder our understanding of this enigmatic process. Despite an increasingly better molecular understanding of XCU, many questions remain unanswered: namely, which mechanisms confer specificity to the upregulation (i.e. how do they target X-linked genes), and whether all X-linked genes or a subset need to be upregulated? Of note, alternative mechanisms have been reported that have (potentially) allowed compensation the hemizygosity of X-linked genes during sex chromosome evolution; these include the downregulation of autosomal genes that are partners of X-linked genes (Julien et al., 2012), retention of a functional gene copy on the Y chromosome (Bellott et al., 2014; Cortez et al., 2014), duplication of genes on the X (Julien et al., 2012), and relocalisation of proto-Y genes to autosomes (Carelli et al., 2016; Hughes et al., 2015; Potrzebowski et al., 2008). Interestingly, in rodents that have lost the Y chromosome completely, some ‘Y-linked’ genes (presumably the dosage-sensitive ones) are found on the X or autosomes (Arakawa et al., 2002; Kuroiwa et al., 2010; Mulugeta et al., 2016).

### X-chromosome inactivation: convergent evolution in therian mammals

Based on several genetic studies and observations in mammals, Mary Lyon put forward the idea of X-chromosome inactivation in 1961, by proposing that the dark-staining X chromosome in female somatic cells (Barr and Bertram, 1949; Ohno et al., 1959) was inactivated (Lyon, 1961). The 60th anniversary of her seminal proposal was celebrated recently (Moyano Rodriguez and Borensztein, 2023). Insightfully, Lyon also anticipated that this inactive X could be ‘either paternal or maternal in origin in different cells of the same animal’ (what is referred to as random XCI, true for placental mammals but not for marsupials) and that it occurred early in embryonic development (Lyon, 1961). A truly epigenetic process, XCI is heritable through mitosis and can be reversed (X-chromosome reactivation), which happens in specific developmental stages and pathological contexts (Panda et al., 2020; Spaziano and Cantone, 2021; Talon et al., 2019). How XCI is triggered specifically in XX individuals, how it affects only one X chromosome and the molecular mechanisms that are implicated in the transcriptional silencing of the X, which is accompanied by heterochromatinisation and chromosome refolding, have been recently reviewed elsewhere (Kanata et al., 2024; Keniry and Blewitt, 2023; Loda et al., 2022; Mutzel and Schulz, 2020; Schwämmle and Schulz, 2023). Here, we cover the evolutionary diversity observed in XCI across mammalian species and its implications for our understanding of dosage regulation and compensation.

According to Ohno's hypothesis, XCI was the ‘second step’ needed for X-linked dosage compensation upon differentiation of the mammalian sex chromosomes; XCI evolved in XX individuals to counteract the effects of XCU, which balanced X-linked gene expression to autosomes in XY individuals but created a problem for XX. Often in the field, XCI is mentioned as having evolved to ‘equilibrate X-linked gene expression between the sexes’, an oversight because of course selection does not work on the balance between the sexes, but on the individual (Vicoso and Bachtrog, 2009). Besides being imprecise, such formulation reinforces the idea that compensated X-linked gene expression is expected to be the same between the sexes, although this might not be the case. Instead, it just needs to be compatible with life and reproduction in each sex [‘incomplete but sufficient’ (Gu and Walters, 2017)]. This means, for example, that the X:autosome expression ratio (for each gene) does not have to be exactly the same in XX and XY individuals.

**Fig. 2.**
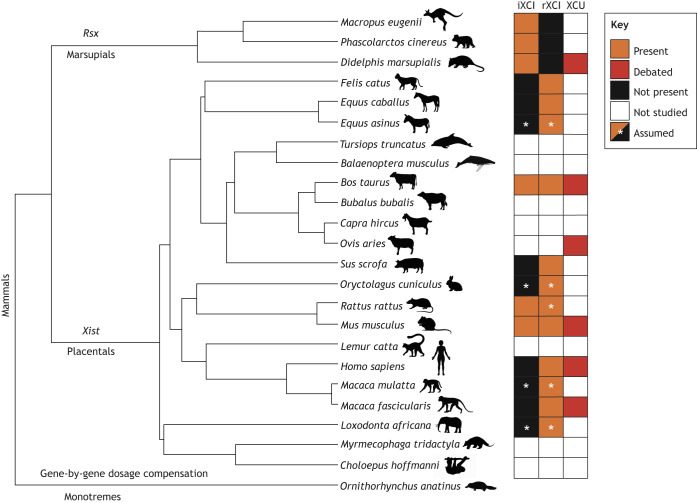
**The evolutionary diversity of dosage compensation mechanisms among mammals.** The phylogenetic tree (generated with timetree.org) displays various mammalian species. On the right, we have indicated what is known regarding X-linked dosage compensation processes in the species presented (iXCI, imprinted X-chromosome inactivation; rXCI, random X-chromosome inactivation; XCU, X-chromosome upregulation). Boxes in orange denote presence, in black denote absence, in red denote conflicting research and in white denote absence of studies. White asterisks indicate that a given process is assumed to be present but has not been formally demonstrated. The pictograms used for the tree are solely for visualisation purposes and might not correspond to specific species. References can be found in Table 1.

Remarkably, XCI is present in both marsupials and placental mammals (Fig. 2), but it appears to have evolved independently in these two lineages (reviewed by Shevchenko et al., 2013). Marsupial and placental XCI do share certain features: relying on the activity of long noncoding RNAs (lncRNAs), having the same functional outcome (silencing of X-linked genes), the inactive X being targeted by H3K27 trimethylation and to the perinucleolar compartment (Mahadevaiah et al., 2009), but their genetic origins are not homologous. At the forefront of orchestrating XCI in placental mammals stands the lncRNA, *Xist*, discovered more than 30 years ago (https://thenode.biologists.com/xist-discovery/discussion/). *Xist* is essential for XCI in mice (Marahrens et al., 1997; Penny et al., 1996), but no *Xist* gene has ever been found in marsupials (Davidow et al., 2007; Deakin et al., 2009; Hore et al., 2007; Okamoto and Heard, 2009; Shevchenko et al., 2007; Waters et al., 2005). The *Xist* gene is proposed to have emerged *de novo* in eutherians, exhibiting remnants traceable to mobile elements spanning diverse classes and to *Lnx3*, a protein-coding gene present in birds and marsupials but that no longer exists in eutherians (Duret et al., 2006; Elisaphenko et al., 2008). In marsupials, XCI is associated with a different lncRNA, *Rsx*. Although not being a sequence homologue of *Xist*, their RNAs share many functional attributes, such as the female-specific expression, the association and ‘coating’ of the inactive X, the activity in *cis*, and a similar protein interactome (Grant et al., 2012; Mahadevaiah et al., 2020; McIntyre et al., 2024). Formal genetic evidence of *Rsx* being essential for marsupial XCI is still lacking; however, expression of *Rsx* in mESCs from an autosomal transgene resulted in gene silencing in *cis* (Grant et al., 2012). It is noteworthy that marsupial XCI is imprinted (it is always the paternal X that is inactivated in XX somatic cells, whereas in placentals XCI is random) and comparatively more incomplete than the Xist-driven process (reviewed by Deakin et al., 2009).

Marsupial and placental XCI are thus a compelling illustration of convergent evolution, driven by two independently evolved lncRNAs that silence the activity of nearly an entire chromosome (McIntyre et al., 2024). Importantly, as mentioned before, the sex chromosomes in marsupials and placentals are homologous, whereas the dosage compensation mechanisms in XX individuals are not; it is unclear whether these were preceded by an older dosage compensation mechanism in the last common ancestor. Some authors have suggested an initial process potentially involving other noncoding RNAs, possibly acting gene-by-gene (as in monotremes, Box 3), which was replaced by chromosome-wide regulation by *Xist* and *Rsx* to facilitate more efficient silencing (Gribnau and Grootegoed, 2012; McIntyre et al., 2024). It is also possible that *Rsx* represents the ancestral regulator, whether acting gene-by-gene or chromosome-wide. Interestingly, a recent study in chicken has shown that a microRNA contributes to the downregulation of Z-linked genes in ZZ individuals, which are upregulated in ZW individuals (Fallahshahroudi et al., 2024 preprint).

### Placental XCI: a diversity of roads leading to random XCI

Investigating XCI in placentals other than the mouse (e.g. human, macaque, rabbit, cow and pig) has revealed evolutionary flexibility in the regulation and dynamics of XCI (Fig. 2). Some of the truths we learnt about XCI with the mouse appear to be more of an exception than the rule in placental mammals, probably representing recently evolved characteristics rather than common ancestral traits – a likely reflection of the extensive evolutionary radiation of rodents (Fabre et al., 2012) and of the mouse genome evolving faster than that of larger mammals due to more generation cycles per unit of time (Svoboda, 2018).

**Fig. 3.**
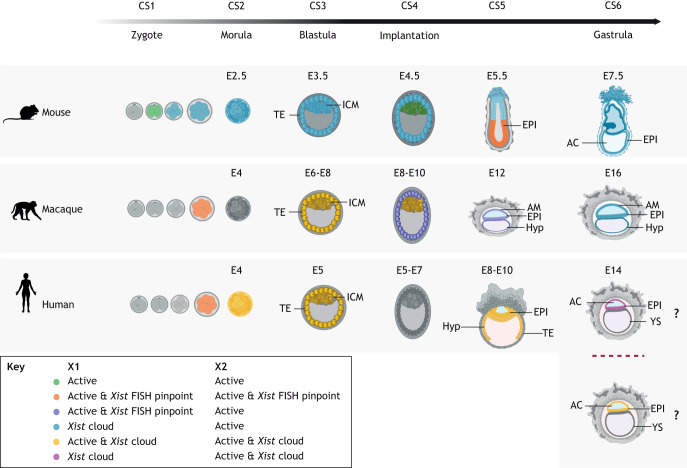
**Colourful development: X-linked states in mice, macaques and humans.** The dynamics of both X chromosomes during development in mice, monkeys and humans are summarised from available studies. Colours represent the status of one X chromosome (X1) or the other (X2), illustrating how XCI patterns during early development are quite variable across species. The ‘active’ status indicates either the expression of one or more X-linked genes assessed using fluorescence *in situ* hybridisation (FISH) or by single-cell RNA sequencing. Schemes inspired by Nakamura et al. (2021), Okamoto et al. (2021), Saiba et al. (2018), Shevchenko et al. (2019) and Zhai et al. (2022).

When does XCI take place during development? In all species examined so far, the random XCI pattern observed in somatic cells is first detected in post-implantation embryos. This corresponds, for example, to embryonic day (E)6-E7 in the mouse and E15-E17 in the macaque, which raises interesting questions regarding the time and extent to which gene dosage compensation is needed (Heard and Rougeulle, 2021; Okamoto et al., 2021). However, it is also important to consider the differences between chronological time and developmental time (Dubansky, 2018; Garcia-Ojalvo and Bulut-Karslioglu, 2023). Even if macaque embryos go through more days without dosage compensation, in terms of ‘developmental time’ dosage compensation appears to be required at a similar stage as in the mouse (Fig. 3).

Not all species, however, reach random XCI in the same way. In the mouse, there is an earlier wave of XCI, which is paternally imprinted, similar to marsupials (another likely example of convergent evolution) and occurs during preimplantation stages (reviewed by Furlan and Galupa, 2022). Murine imprinted XCI is maintained in the extra-embryonic tissues and reverted in the inner-cell mass of the blastocyst, which gives rise to the epiblast cells, in which random XCI occurs some days later. In placental mammals, imprinted XCI is restricted to extra-embryonic tissues and appears to also be present in rat and bovine embryos (Dindot et al., 2004; Magaraki et al., 2019; Wake et al., 1976; Xue et al., 2002; Yu et al., 2020), but absent in human, macaque, rabbit, pig and horse, where extra-embryonic tissues show random XCI (Beckelmann et al., 2012; Goszczynski et al., 2021; Moreira de Mello et al., 2010; Okamoto et al., 2011, 2021; Ramos-Ibeas et al., 2019; Romagnano et al., 1987; Wang et al., 2012; Zou et al., 2019). Which species show imprinted XCI does not appear to be associated with the type of placental structure (Laudon et al., 2024). Instead, it has been linked to earlier zygotic genome activation (ZGA) and faster development (Migeon, 2002), but this does not appear to hold in bovine embryos (Svoboda, 2018). Evolutionary considerations about imprinted XCI have recently been reviewed by Furlan and Galupa (2022).

In the mouse, the expression of *Xist* is tightly coupled to XCI. Intriguingly, this is not the case for other mammalian species. During preimplantation development, *XIST* RNA is detected coating the X chromosome for several days without inducing gene silencing in human, monkey, rabbit and bovine embryos (Okamoto et al., 2011, 2021; Yu et al., 2020). Moreover, *XIST* RNA is detected coating both X chromosomes in XX embryos or even the X chromosome in XY human and macaque embryos for several days (Okamoto et al., 2011, 2021; Vallot et al., 2017). It is still unclear why and how *XIST* RNA accumulation is uncoupled from XCI, and what is the switch/trigger that allows these pre-XCI but *XIST*-associated states to eventually be resolved into random XCI in XX embryos and no XCI in XY embryos. Nevertheless, in human embryos, *XIST* presence might not be without consequences: it coincides with the downregulation of X-linked gene expression, which has been termed X-chromosome ‘dampening’ (XCD) (Petropoulos et al., 2016). Dampening does not appear to be present in other primates, such as the macaque or marmoset (Cidral et al., 2021; Okamoto et al., 2021) and remains contested in human (Moreira de Mello et al., 2017; Mandal et al., 2020). Recently, it has been shown in human ESCs that deletion of *XIST* leads to derepression of X-linked expression (suggesting that *XIST* is responsible for XCD) and that SPEN, the transcriptional repressor that is essential for initiating gene silencing during XCI (Dossin et al., 2020), is also involved (Alfeghaly et al., 2024; Dror et al., 2024). Again, what prevents the expression of XIST and recruitment of SPEN to lead to full XCI remains unknown.

### New insights into the regulation of *Xist* across placental species

The regulation of *Xist* preceding random XCI also shows species-specific variations, at least, as evaluated by studies on cultured mouse and human cells. *Xist* is embedded in a regulatory landscape with many other noncoding loci (reviewed by Luchsinger-Morcelle et al., 2024), some of which evolved via pseudogenisation from protein-coding genes along with *Xist* (Chureau et al., 2002; Duret et al., 2006). Whether *Tsix*, which runs antisense to *Xist* and is essential for XCI regulation in mice, is as important in other species is an old debate (reviewed by Galupa and Heard, 2018). For now, genetic evidence to support or disprove such a role is still missing. More recently, Claire Rougeulle's lab has spearheaded functional analyses in primate ESCs of some of the noncoding neighbours of *XIST*, the *JPX* and *FTX* loci that, in mouse, are important positive regulators of *Xist* and random XCI (Furlan et al., 2018; Gjaltema et al., 2022; Sun et al., 2013; Tian et al., 2010). Interestingly, *FTX* functions are not conserved in human (Rosspopoff et al., 2023), and *JPX* is also a major regulator of *XIST* regulation in human but not in macaque or marmoset (Cazottes et al., 2023 preprint; Rosspopoff et al., 2023). Yet, between human and mouse there are differences in how *Jpx*/*JPX* regulates *Xist*/*XIST*. In mouse, the *Jpx* RNA mediates *Xist* regulation, at the post-transcriptional level; in human, the *JPX* RNA is dispensable for XIST regulation. Rather, it is *JPX* transcription that is required for proper *XIST* expression, in *cis*, probably via influencing RNA polymerase II recruitment to the *XIST* promoter (Rosspopoff et al., 2023). In macaque and marmoset ESCs, no indications have been found of ongoing transcription at syntenic positions of *Tsix*, *Linx* or *Xite* (also known as *Rr18*) (Cazottes et al., 2023 preprint), other noncoding loci recognised as significant *Xist* repressors in mice (Galupa et al., 2020; Hierholzer et al., 2022; Lee, 2000; Lee and Lu, 1999; Ogawa and Lee, 2003; Sado et al., 2001).

Unravelling the diverse mechanisms and regulatory strategies governing XCI across eutherian mammals sheds light on the intricate evolutionary dynamics of this dosage compensation process. As stated by Okamoto and colleagues, the existing diversity ‘probably reflects the fact that developmental processes are constantly changing during evolution and that the regulation of processes such as XCI have to display substantial plasticity to accommodate these changes’ (Okamoto et al., 2011).

### A dance of upregulation and inactivation: evolutionary and developmental dynamics of XCU and XCI

Despite the sequential narrative of XCU and XCI in Ohno's hypothesis, they have had to evolve rather ‘simultaneously’ (and potentially influencing each other), as genes were lost from the proto-Y chromosome, creating a need for dosage-compensation mechanisms. It remains unclear how XCU and XCI evolved per se and in relation to each other, which is especially intriguing considering that XCU appears to operate on a gene-by-gene basis, whereas XCI is a chromosome-wide mechanism. The latter was perhaps not the case in the initial stages of sex-chromosome differentiation (Disteche, 2016; Gribnau and Grootegoed, 2012), in which dosage compensation in XX individuals might have happened on a gene-by-gene basis, rather resembling what is observed in present-day monotremes (Box 3).

Although the evolutionary dynamics remain enigmatic, progress has been made recently regarding the developmental dynamics of XCU and XCI (Fig. 1B). Based on allele-resolved single-cell RNA sequencing (scRNA-seq) of mouse embryos and embryonic stem cells, XCU has been proposed to occur on one of the two X chromosomes while the second one is undergoing XCI (Lentini et al., 2022). In the authors' words, ‘a flexible process that tunes RNA synthesis proportionally to the output of the second X allele across developmental states’ (Lentini et al., 2022). For this reason, the authors called it an ‘elastic’ process of dosage compensation, as opposed to the X chromosome(s) being constantly upregulated (before XCI) or upregulated as a single developmental event. In XY embryos, XCU is established upon ZGA and maintained throughout development, whereas in XX embryos XCU accompanies XCI: it occurs initially upon ZGA along with imprinted XCI, but is then reversed in embryonic lineages as the inactive X reactivates, and established again along with random XCI (Lentini et al., 2022) (Fig. 1B). Another recent study has also found that XCU is dynamically linked to random XCI (Naik et al., 2022). XCU thus happens in response to imbalanced X dosage, which has been further supported by reanalysis of allele-resolved scRNA-seq from *Xist* knockout embryos (Borensztein et al., 2017); in the absence of XCI, no XCU was initiated (Lentini et al., 2022). Overall, these findings (especially the timings at which XCU occurs) contrast with observations made in some previous studies (Table 1), probably because these previous studies did not take into account the allelic origin of X-linked gene expression, which can be confounded by processes such as XCI and ZGA (e.g. XCI and XCU on opposing alleles may cancel out if analysing only cumulative RNA level). Allele-resolved scRNA-seq analyses during macaque embryogenesis revealed similar findings as in mice, showing that in XX embryos XCU occurs along with or after XCI, whereas in XY embryos it takes place progressively from the first stages analysed (Okamoto et al., 2021).

Such elastic XCU implies a dosage-sensing mechanism coupling XCU and XCI in XX embryos. The same authors (Lentini et al., 2022) proposed that this could be achieved through a progressive shift of transcription factors to the active X from the inactive X territory, from which they are excluded as the inactive-X repressive compartment is formed (Chaumeil et al., 2006; Collombet et al., 2023). How XCU might be coupled to ZGA in XY embryos is less clear.

## Which X-linked genes need to be dosage-compensated?

The assumption underlying the importance of dosage-compensation mechanisms is that their absence leads to detrimental phenotypes. So far it is not possible to manipulate XCU to test its importance, given that we still know so little about its mechanisms, but XCI instead can be abolished by knocking-out its major regulator. Failure to undergo XCI upon *Xist* deletion during early mouse development has revealed genome-wide changes in gene expression and embryonic lethality due to defects in extra-embryonic tissues (Borensztein et al., 2017; Marahrens et al., 1997; Mugford et al., 2012). Such a phenotype is likely an additive (or synergistic) result of many X-linked genes not being dosage-compensated. But throughout the evolution of mammalian sex chromosomes, as it is likely that genes on the proto-X were dosage-compensated gradually while genes on the proto-Y were being gradually lost, did all genes on the X have to be dosage-compensated? Has the evolution of dosage-compensation mechanisms been ‘driven predominantly by a need to equalise overall X-linked and autosomal expression levels’ or do ‘transcript levels of key individual genes exert the major selection pressure’ (Lin et al., 2007)? We know that, for many genes, heterozygous mutations are well tolerated. Other genes, on the contrary, show haploinsufficiency (deletion intolerance) or triplosensitivity (duplication intolerance) (Collins et al., 2022). It therefore appears to be reasonable that dosage-sensitive genes on proto-sex chromosomes have been the main drivers for the emergence of dosage-compensation mechanisms during sex-chromosome evolution. Supporting this notion, Pessia and colleagues have shown that, for X-linked genes presumably more dosage-sensitive (coding for members of large protein complexes, with ≥7 proteins), dosage compensation is more prevalent. Such genes showed higher expression ratios between the X and autosomes when compared with others coding for smaller protein complexes (Pessia et al., 2012), suggesting, therefore, that dosage compensation for such genes is more required.

Dosage-sensitive genes typically encode factors for which there are stoichiometry constraints, such as subunits of large complexes, transcription factors, members of signal transduction pathways or microRNAs (Basilicata and Keller Valsecchi, 2021; Birchler and Veitia, 2007; Desvignes et al., 2021; Meunier et al., 2013; Veitia and Birchler, 2022). Based on curated genomic data (Blake et al., 2021) and gene ontology analysis (Ashburner et al., 2000; The Gene Ontology Consortium, 2021), we have determined that the mouse X chromosome harbours 92 miRNA loci and 507 genes involved in processes related to signalling and/or transcription and/or that code for components of protein complexes. Which ones correspond *de facto* to dosage-sensitive genes remains to be determined; unfortunately X-linked genes (and Y-linked) are often excluded from genome-wide analyses, as was the case for the recent catalogue of human dosage-sensitive genes (Collins et al., 2022). Some authors have proposed that the X is depleted of dosage-sensitive genes (the ‘insensitive X hypothesis’), based on analyses that determine that X-linked genes are less dosage-sensitive than autosomal genes, and that dosage-sensitive housekeeping genes are preferentially located on autosomes (Chen et al., 2020; Lin et al., 2012; Yang and Chen, 2019). This could reflect the initial gene content of the proto-sex chromosomes; in fact, some chromosomes are thought to be better suited to become sex chromosomes, based on their gene content, and sex chromosomes do tend to originate from autosomes that are overall insensitive to dose changes (Bachtrog et al., 2011; Disteche, 2016; Livernois et al., 2012). Interestingly, the emergence of dosage-compensation mechanisms can in turn influence the evolution of the sex chromosomes themselves in terms of gene content (Box 4).

**Box 4. Consequences of dosage compensation for the evolution of the X chromosome**

Dosage-sensitive functions have been proposed to drive the preservation of genes on sex chromosomes (Bellott and Page, 2021; Naqvi et al., 2018), and the mammalian X does have a non-random gene composition: it is enriched in genes with brain-related and reproduction-related functions (Graves et al., 2002; Leitão et al., 2022). Of note, the human X is enriched for male-specific but not female-specific genes (Lercher et al., 2003). Although male-specific genes have likely started accumulating on the X upon sex-chromosome differentiation (Julien et al., 2012), the enrichment in brain-related genes is thought to reflect the ancestral state of the proto-sex chromosomes (Kemkemer et al., 2009). The gene content of the X chromosome shows a remarkable degree of conservation across therian mammals, unparalleled by any autosome, and it is also the chromosome in mammals that retains the highest synteny (or gene order, linkage or collinearity), as well as conserved recombination patterns (Deakin et al., 2013; Kim et al., 2017; Lewis et al., 2002; Li et al., 2019; Murphy et al., 2005; Nadeau, 1989; Sinha and Meller, 2007). This suggests that both the evolution of synteny and gene content on the X are constrained, and this has been attributed to selective pressures aimed at preserving dosage compensation and, in particular, X-inactivation (Delgado et al., 2009; Ohno, 1967). Another explanation has recently emerged: comparing the genome assemblies of cat, pig, human and mouse, Brashear and colleagues have proposed that the selective constraints are due to the three-dimensional genomic architecture of the X that is necessary to fold the inactive X chromosome in its two typical mega-domains (Brashear et al., 2021). This would imply that such folding has a crucial function, which remains as yet unconfirmed.

This does not exclude that particularly dosage-sensitive genes on the X have favoured the evolution of dosage-compensation mechanisms. For example, the X harbours the gene *SMC1A*, a subunit of the cohesin complex, which mediates sister chromatid cohesion, homologous recombination and DNA looping. Other cohesin subunits, such as Nipbl, are haploinsufficient (Mills et al., 2018). Heterozygous mutations in *SMC1A* itself have also been associated with haploinsufficiency underlying Cornelia de Lange syndrome (Deardorff et al., 2007; Musio et al., 2006). Another example of highly likely dosage-sensitive X-linked genes are *MED14* and *MED12*, both subunits of the mediator complex, which play essential functions in eukaryotic transcription; higher levels of MED14 as a result of abnormal X-reactivation have been associated with impairment of mammary stem cell differentiation and increased tumorigenicity (Richart et al., 2022). Importantly, the mammalian X chromosome consists of a mix of ancestral genes and more recently acquired ones, and it has been suggested that these two gene groups might have different compensation requirements and potentially involve distinct regulatory mechanisms (Deng et al., 2013).

## Conclusion

Understanding the regulation and compensation of mammalian gene dosage has clearly provided us with many new insights into development and evolution, which extends our understanding of physiology and pathology. For example, *XIST* transgenics has gained interest as a possible therapeutic tool for chromosome dosage disorders, such as Down syndrome (Gupta et al., 2024; Moyer et al., 2021). An aspect that we overlooked in this Review is that a subset of X-linked genes escapes XCI (Carrel et al., 1999), meaning they show biallelic expression in XX individuals. For some of these genes this translates to higher dosage compared with XY individuals, whereas for others this could be the means for dosage compensation, as they have homologues in the Y chromosome (Box 2). These escaping genes (including *XIST*) underlie sex-biassed susceptibility to certain diseases, such as autoimmune diseases (Dou et al., 2024; Forsyth et al., 2024; Hagen et al., 2020; Souyris et al., 2018; Youness et al., 2021). Interestingly, recent studies have shown how expression from the inactive X can modulate gene expression from the active X and autosomes (San Roman et al., 2023, 2024; Topa et al., 2024; Zhang et al., 2024).

Many important questions remain unanswered. As incomplete dosage compensation is well tolerated among some vertebrates, such as birds, what makes therian mammals (especially placental mammals) more sensitive to dosage differences, underlying the need for tight, chromosome-wide dosage-compensation mechanisms? Could this be related to constraints on the placenta and/or other extra-embryonic tissues? It is interesting to note that early embryonic lethality in mouse mutants is very often associated with severe placental malformations (Perez-Garcia et al., 2018). Another important open question is the extent to which the dosage sensitivity of a given gene will depend on the cell type or tissue where it is expressed.

New progress will certainly be achieved with the continuous improvement of technologies that allow us to quantify gene expression beyond transcription levels, such as quantitative proteomics (Schubert et al., 2017), or to quantitatively modulate gene expression (Ma et al., 2024; Naqvi et al., 2023; Noviello et al., 2023). Additionally, new embryonic systems *in vitro* hold promise to enable the exploration of a higher number of mammalian species (Handford et al., 2024; Lázaro et al., 2024), as well as to allow us to start functionally testing hypotheses about the dynamics and timing of dosage compensation. We look forward to the upcoming exciting times for dosage compensation research.
